# Biosynthesis of micro‐ and nanocrystals of Pb (II), Hg (II) and Cd (II) sulfides in four *Candida* species: a comparative study of *in vivo* and *in vitro* approaches

**DOI:** 10.1111/1751-7915.12485

**Published:** 2017-01-16

**Authors:** Mayra Cuéllar‐Cruz, Daniela Lucio‐Hernández, Isabel Martínez‐Ángeles, Nicola Demitri, Maurizio Polentarutti, María J. Rosales‐Hoz, Abel Moreno

**Affiliations:** ^1^ Departamento de BiologíaDivisión de Ciencias Naturales y ExactasCampus GuanajuatoUniversidad de GuanajuatoNoria Alta S/N, Col. Noria AltaC.P. 36050GuanajuatoMéxico; ^2^ Departamento de Química de BiomacromoléculasInstituto de QuímicaUniversidad Nacional Autónoma de MéxicoAv. Universidad 3000Ciudad UniversitariaCiudad de México04510México; ^3^ Elettra – Sincrotone TriesteS.S. 14 km 163.5 in Area Science Park34149Basovizza – TriesteItaly; ^4^ Departamento de QuímicaCentro de Investigación y de Estudios Avanzados del I.P.N.Apdo. Postal 14‐74007000México, D.FMéxico

## Abstract

Nature produces biominerals (biogenic minerals) that are synthesized as complex structures, in terms of their physicochemical properties. These biominerals are composed of minerals and biological macromolecules. They are produced by living organisms and are usually formed through a combination of chemical, biochemical and biophysical processes. Microorganisms like *Candida* in the presence of heavy metals can biomineralize those metals to form microcrystals (MCs) and nanocrystals (NCs). In this work, MCs and NCs of PbS, HgS or HgCl_2_ as well as CdS are synthesized both *in vitro* (gels) and *in vivo* by four *Candida* species. Our *in vivo* results show that, in the presence of Pb^2+^, *Candida* cells are able to replicate and form extracellular PbS MCs, whereas in the presence of Hg^2+^ and Cd^2+^, they did synthesize intercellular MCs from HgS or HgCl_2_ and CdS NCs respectively. The MCs and NCs biologically obtained in *Candida* were compared with those PbS, HgS and CdS crystals synthetically obtained *in vitro* through the gel method (grown either in agarose or in sodium metasilicate hydrogels). This is, to our knowledge, the first time that the biosynthesis of the various MCs and NCs (presented in several species of *Candida*) has been reported. This biosynthesis is differentially regulated in each of these pathogens, which allows them to adapt and survive in different physiological and environmental habitats.

## Introduction

Natural gemstones (sapphires, diamonds, emeralds, rubies) and minerals (calcite, quartz, pyrite, galena), grown under Earth's surface for millions of years, are remarkable for their shining beauty, fascinating colours, extraordinary geometry and shape. These extraordinary characteristics have enticed men into wearing them as luxurious ornaments, amulets or even into using them in ancient medicine or in high‐technology applications as we do nowadays. However, the most extraordinary minerals, in terms of technological applications and biomedical properties, are those forming complex structures that appear in biological systems. These are called biominerals, as they are composed of minerals and biological macromolecules and are produced by living organisms in the formation of bones, teeth, stromatolites, eggshells, nacre shells, among others. Biomineralization studies the properties, structure and formation of inorganic solids deposited in biological systems, and it is also relevant to the Earth's environmental and evolution processes on practically all scales. The impact of biomineralization is recorded on a global scale and stretches far back in the history of life (Mann and Weiner, 1999). There is one approach that helps to emulate nature in growing these extraordinary minerals. This approach is called the crystal growth in gel technique. This gel technique favours the growing of crystals as instantaneous mineralogy. This mean that, where nature takes millions of years to grow those beautiful minerals, we will be able to do the same in just a few months, weeks or even days (Henisch and Garciaruiz, 1986a,b; Garciaruiz, 1991). On the other hand, the most exciting crystal growth method of technologically and biomedical important materials in the form of microcrystals (MCs), nanocrystals (NCs) or as nanoparticles (NPs) are those biogenic minerals produced by different microorganisms from bacteria to fungi. However, these minerals of biological origin are sometimes difficult to grow *in vitro* that is probably why they have never being synthesized in a laboratory (Mann and Ozin, 1996). It is through biomineralization that these organisms synthesize MCs, NCs or nanoparticles (NPs). These MCs or NCs were reported to have properties of semiconductor quantum crystallites (Williams *et al*., 1996). Microcrystals and nanocrystals (MNCs) are usually formed through a combination of chemical, biochemical and biophysical processes. Biosynthesis of MCs or NCs by microorganisms is therefore a great alternative to conventional systems of crystal growth, as MCs and NCs produced through biosynthesis are relatively easy to reproduce and more stable compared with those synthesized *in vitro* (Inouye *et al*., 1982; Borrelli *et al*., 1987; Herron *et al*., 1989; Williams *et al*., 1996). In addition, stable MCs and NCs have several useful applications in different areas. In nanotechnology, for instance, they can be used as sensors (Kowshik *et al*., 2002; Krumov *et al*., 2007), they can also be used as quantum semiconductors (Williams *et al*., 1996), as markers, in biological systems (Krumov *et al*., 2007) and also in health care, cosmetics, chemical industries and space (Korbekandi *et al*., 2014), as well as in environmental remediation due to their ability to sequester and detoxify intracellular cadmium ions (Dameron *et al*., 1989). Other studies have reported that yeasts such as *Saccharomyces cerevisiae* (Prasad and Jha, 2010), *Schizosaccharomyces pombe* and *C. glabrata* are good producers of NCs in the presence of heavy metals and peptides with the general structure (γ‐Glu‐Cys)_n_‐Gly; Dameron *et al*., 1989). Even though *C. glabrata* has been used in the biosynthesis of CdS NPs, not other *Candida* species so far have been reported capable of biosynthesizing NCs like this yeast, nor has it been reported whether these microorganisms are capable of producing MCs and/or NCs in the presence of other chemical elements such as mercury (II) or lead (II). MCs formed in the presence of these elements are of special interest, because they can be used to monitor the concentration of these heavy metals in contaminated environments (Pham *et al*., 2015). Furthermore, it has also been reported that HgS NCs are technologically important materials (Jeong *et al*., 2014). However, their instability has hampered their usefulness (Yang *et al*., 2015). NCs chemically synthesized from Cd:HgS/CdS have proved to be stable and highly fluorescent compared with the HgS NCs also obtained by chemical synthesis (Yang *et al*., 2015). Other research groups have used other synthesis strategies for AgS NCs, but all with a high degree of complexity (Han *et al*., 2014; Jeong *et al*., 2014). The PbS NCs were chemically synthesized, and like HgS NCs, the chemical synthesis was also complex and needed to be well controlled *in vitro* (Lim *et al*., 2014; Lee *et al*., 2016). Obtaining stable MCs or NCs from PbS is of special interest due to their widespread use in photo detectors and solar cells (Lim *et al*., 2014; Ko *et al*., 2016; Lee *et al*., 2016). Although NCs have been synthesized *in vitro* in the presence of mercury (II) and lead (II), our working group is interested in synthesizing MCs or NCs of PbS, HgS and CdS not just *in vitro* but also *in vivo*, using gels and microorganisms of *Candida* respectively. This method will allow us to obtain MNCs with high reproducibility and stability, thus enhancing their number of applications in future.

The goal of this study was to evaluate the formation of MCs or NCs of lead (II), mercury (II) and cadmium (II) sulfide *in vitro* (gels) and *in vivo* in four species of *Candida* (*C. albicans, C. glabrata, C. krusei* and *C. parapsilosis*).

## Results and discussion

### Growth of *Candida* is inhibited in the presence of Hg^2+^ and Cd^2+^, but not in the presence of Pb^2+^

**Figure 1:**
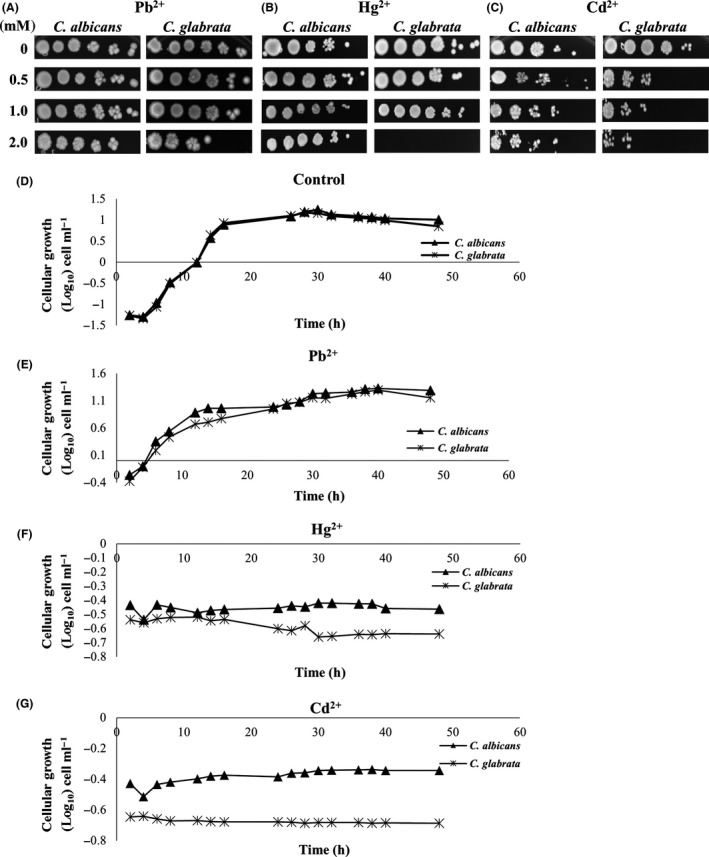
Response of *Candida* species to different metals. (i) Susceptibility tests: cultures of the two *Candida* species were diluted to an OD_600 nm_ 0.5 with sterile deionized water. Aliquots of dilutions were incubated at 28°C in the presence of the indicated concentrations of (A) Pb^2+^, (B) Hg^2+^ or (C) Cd^2+^. After 90 min, water was removed by low‐speed centrifugation and the corresponding cell pellets were resuspended in sterile deionized water to an OD_600 nm_ 0.5. (ii). Growth curves of *C. albicans* and *C. glabrata*. (D) *Candida* cells were not exposed to heavy metals (controls). Cells were exposed at a concentration of 1.0 mM (E) Pb^2+^, (F) Hg^2+^ or (G) Cd^2+^. From each culture in the presence of metal, an aliquot was taken every 2 h, over 48 h, and the OD_600 nm_ was measured in a spectrophotometer. The log_10_ of the OD_600 nm_ was plotted against time in hours.

In order to determine how *C. albicans*,* C. glabrata*,* C. krusei* and *C. parapsilosis* respond to Pb^2+^, Hg^2+^ or Cd^2+^, as the first step in the evaluation of their capacity to synthesize MCs or NCs, the cells were exposed to each of these metals. We observed that these four species of *Candida* were able to replicate in the presence of Pb^2+^. However, no growth/replication was observed in the presence of Hg^2+^ or Cd^2+^. In order to corroborate that the growth is indeed affected by the presence of Hg^2+^ or Cd^2+^, but not for Pb^2+^, we had to test the susceptibility of these yeasts through serial dilutions and growth curves. For the purpose of the susceptibility testing, cells were placed in the presence of metals at different concentrations, for 90 min, as described in the methods section. A representative figure of the results obtained with two species *Candida* is shown (see Fig. 1A–C). The cells of *C. albicans*,* C. glabrata*,* C. krusei* and *C. parapsilosis* in the presence of Pb^2+^ are able to withstand up to 2.0 mM of the metals (Fig. 1A, Table S1). *C. albicans* cells can grow (in the presence of Hg^2+^) up to 2.0 mM, while *C. glabrata*,* C. krusei* and *C. parapsilosis* can only grow up to 1.0 mM in the presence of this metal (Fig. 1B, Table S1). In the presence of Cd^2+^, the cell growth in the four species of *Candida* is observed up to 2.0 mM, but this growth is considerably affected compared with control cells (Fig. 1C, Table S1). Altogether, these tests show that the four species of *Candida* are able to withstand 1.0 mM of Pb^2+^, Cd^2+^ or Hg^2+^ for 90 min. Although all *Candida* species are able to grow at a concentration of 1.0 mM of any of the three metals evaluated, the ability of any *Candida* species to withstand a higher concentration of these metals will depend on the adaptability of each of these yeasts to the different physiological niches in order to survive in the host (Serrano‐Fujarte *et al*., 2015). In this regard, each species of *Candida* was reported to respond differentially whether they were in the presence of different azole antifungals or in the presence of different reactive oxygen species (ROS; Cuellar‐Cruz *et al*., 2008; Ramirez‐Quijas *et al*., 2015; Serrano‐Fujarte *et al*., 2015). This indicates that the differential resistance to toxic metals may be related to the mechanism of adaptation that each *Candida* species develops for surviving in the environment, even though these yeasts can withstand a concentration of 1.0 mM of any of the analysed metals for a short period (Fig. 1A–C). The most interesting thing here is to find out whether the cells will be able to duplicate themselves. For this purpose, we established growth curves for each of the yeast, from 0 to 48 h, in the presence of each of these four cations, and aliquots were taken manually every two hours. As seen in the presence of Pb^2+^, both *C. albicans* and *C. glabrata* duplicated from the exponential phase up to the stationary phase (Fig. 1E). The same behaviour was observed for *C. krusei* and *C. parapsilosis* (data not shown). These results indicate that apparently, Pb^2+^ does not affect cell growth of *Candida* (Fig. 1D, E). However, cells of the four species of *Candida* did not replicate in the presence of Hg^2+^ or Cd^2+^ compared with that replication of control cells (Fig. 1D, F, G).

These data show that even when the three metals are divalent cations and could act in the same way at the cellular level, *Candida* species have developed a selective detoxification mechanism for each cation, which allowed them to survive in the various habitats to which they have had to adapt. Most identified species of *Candida* are prevalent in rich soil and aquatic habitats that have been contaminated with heavy metals (Hagler and Mendonca‐Hagler, 1981; Suihko and Hoekstra, 1999; Lopez‐Archilla *et al*., 2004). *C. albicans*,* C. glabrata*,* C. krusei* and *C. parapsilosis* are opportunistic human pathogens, which can be isolated from these habitats or from the bloodstream or from any of the organs in human host. Therefore, the fact that *Candida* species are able to withstand up to 2.0 mM of Pb^2+^ (Fig. 1A) and are normally able to duplicate in the presence of this metal (Fig. 1E) compared with Hg^2+^ or Cd^2+^ (Fig. 1F, G) indicates that these yeasts, in both natural habitats and in the human body, are mainly exposed to Pb^2+^. It is this exposure to Pb^2+^ that makes them to develop selective mechanisms to resist this metal.

Other studies have reported that in yeast, the selectivity and capacity of metal uptake depend on cell age, composition of the growth medium, contact time, pH, temperature (Bishnoi and Garima, 2005) and the composition of its cell wall (CW). In *Candida,* the CW retains the metal through interaction with amino, hydroxyl, phosphate, sulfhydryl and carboxyl groups, forming a coordinated covalent bond. Pb^2+^ has high affinity for sulfhydryl groups, by competing with calcium, and tends to replace Zn^2+^ in enzymes such as HIV nucleocapsid protein (HIV‐CCHC) that can cause the complete inhibition of these sulfhydryl groups (Payne *et al*., 1999). It was also observed that lead has the ability to coordinate with nucleic acids (Da Costa and Sigel, 2000). Hg^2+^ was reported to be preferentially methylated by microorganisms forming methylmercury (CH_3_)_2_Hg or CH_3_Hg^+^. Any of these alkylmercury species has a high affinity to form covalent bonds with sulfur (Chwastowska *et al*., 1999). This chemical behaviour explains most of the biological properties of mercury. The affinity for sulfhydryl groups promotes replacement of some metal ions such as Zn^2+^ and Cu^2+^ in various enzymes. Moreover, mercury is also coordinated with relative ease to phosphate groups and heterocyclic bases in nucleic acids (Onyido *et al*., 2004). Cd^2+^ like Pb^2+^ and Hg^2+^ also binds sulfhydryl groups, which inhibits the activity of sensitive enzymes. Interestingly, Cd^2+^ and Hg^2+^ cause considerable oxidative stress (OS), which is, in many cases, the basis of cell genotoxicity (Luo *et al*., 1996; Kwak *et al*., 2003). These metals can react with molecular oxygen to generate bis‐glutathione (GS‐SG), the cation of metal and hydrogen peroxide (Kachur *et al.,* 1998a). Because reduction of GS‐SG requires the participation of NADPH and because the metal cations are linked immediately to other molecules of GSH, cations of heavy metals cause considerable OS (Xiang and Oliver, 1998; Jacob *et al*., 2001). As reservoir of cysteine, glutathione is an important antioxidant system in the cell (Elskens *et al*., 1991). It is also involved in metabolic processes such as cell communication, metal transport and in the regulation of the redox state of proteins for degradation processes and folding (Meister, 1995; Anderson, 1997; Pastore *et al*., 2003). Due to the non‐availability of glutathione, the enzymatic antioxidant mechanisms cannot detoxify the ROS generated during OS, and thus, ROS damages cellular biomolecules such as nucleic acids, lipids and proteins (Klaunig *et al*., 1998). Metallothioneins belong to a group of proteins that play an important role in the toxicity of mercury and cadmium. They are responsible for protecting cells from these metals (Nordberg and Nordberg, 2000). However, we have found that when *Candida* cells are in OS, both the synthesis of metallothioneins and the synthesis of the antioxidant enzyme systems (catalase, superoxide dismutases, glutathione peroxidases, thioredoxins, glutaredoxins) stop. This damages the DNA, and therefore, just the cellular repairing systems alone will not suffice to repair it (Hoeijmakers, 2001; Jin *et al*., 2003; McMurray and Tainer, 2003). For this reason, our results suggest that possibly *Candida* species are not able to replicate in the presence of Hg^2+^ or Cd^2+^ (Fig. 1F, G). This is not the case for Pb^2+^ cations, probably because they do not generate large concentrations of ROS and cannot be detoxified by the yeast's antioxidant mechanisms (Fig. 1E).

### *Candida* species form extracellular crystals with Pb^2+^ and intracellular crystals with Hg^2+^ or Cd^2+^

**Figure 2:**
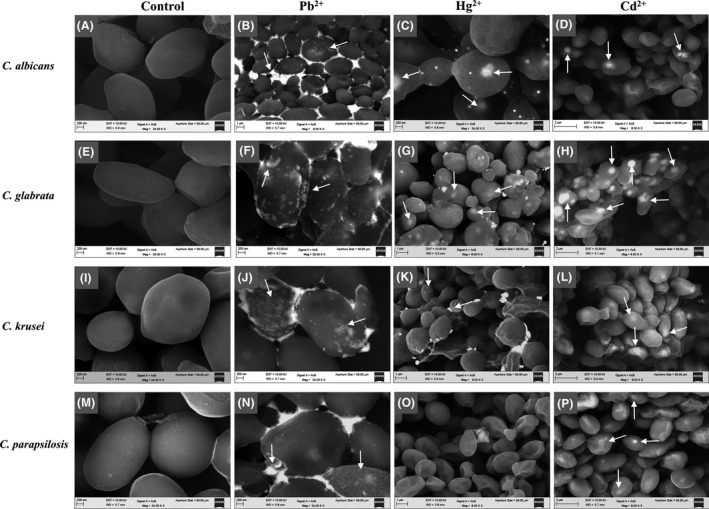
Formation of extracellular and intracellular micro‐ or nanocrystals by *Candida* species in the presence of Pb^2+^, Hg^2+^ or Cd^2+^. *Candida* cells were exposed to the different metals and analysed by SEM as described in the previous methods section. Control cells (A, E, I, M), treated with Pb^2+^ (B, F, J, N), with Hg^2+^ (C, G, K, O) or with Cd^2+^ (D, H, L, P). Scale bar is included in each photomicrograph. Arrows indicate the extracellular and intracellular micro‐ and nanocrystals formed in the treated cells with respect to the control cells. All crystals below 1 *μ*m in size are considered nanocrystals and larger to this value are considered microcrystals.

In order to assess whether the cellular structure of *Candida* is modified in the presence of Pb^2+^, Hg^2+^ or Cd^2+^ (so that the cells cannot be replicated, but can still survive), these four species of *Candida* were grown in the presence of 1.0 mM of each of three metals, which were visualized using scanning electron microscopy (SEM). The most representative images were selected from a large collection and are shown in Fig. 2. Photomicrographs revealed the formation of extracellular and intracellular micro‐ or nanocrystals by *Candida* species in the presence of Pb^2+^, Hg^2+^ or Cd^2+^. The extracellular crystals were observed attached to the cell wall (CW), considering that the CW is the outermost structure of *Candida* and the first to interact with heavy metals. Meanwhile, intracellular crystals were observed like lights inside the yeast. All crystals below 1 *μ*m in size are considered nanocrystals (NCs) and larger to this value are considered microcrystals (MCs).

As in the presence of Pb^2+^, *C. albicans, C. glabrata, C. krusei* and *C. parapsilosis* produced mainly extracellular MCs, which were attached to the CW (Fig. 2B, F, J, N). The synthesis of extracellular MCs of lead may be due to the mechanisms developed by *Candida*, which permitted to form these MCs in the CW (Fig. 2B, F, J, N) and thus protect the biomolecules of the cell membrane, so that its cell viability is not affected (Fig. 1E). One of these mechanisms involves biosorption of Pb^2+^ by the CW, as this is the site where metal retention is performed by a physicochemical interaction with amino and hydroxyl groups of the chitin in the CW, such as phosphate, sulfhydryl and carboxyl groups. This interaction results in the formation of a coordinate covalent bond, in which the metal ion acts as a central atom with empty orbitals capable of accepting electron pairs (Aslangul *et al*., 1972). Another mechanism that explains why lead MCs are formed on the outside of the CW is the extracellular precipitation mechanism, in which the microorganism (in this case *Candida* in the presence of Pb^2+^) activates the synthesis of extracellular components of low molecular weight peptides that act as chelating agents (Baldrian, 2003). Another chelating system or immobilization of the metals may be compounds such as oxalates, sulfides and organic polycarboxylic acids (Gadd and White, 1993; Sierra‐Alvarez, 2007). Thus, the chelating agents form a complex or immobilize the soluble metal ions to insoluble compounds, decreasing the bioavailability of these metals, but increasing their tolerance. A third mechanism by which *C. albicans* could synthesize lead MCs in the CW has been reported elsewhere in fungi such as those of *Pleurotus ostreatus, Phanerochaete chrysosporium* and *Trametes versicolor*. This mechanism produced an extracellular hyphal sheath mainly composed of polysaccharides (β‐1,3 and β‐1,6 glucans) that trap metal ions, thereby providing a barrier against the metal. Another way in which these yeasts adapt to different conditions is through a mutation in the genes that encode any of the lead transport systems. By doing so, they do not bond with this cation and the cells will be able to tolerate this metal (Fig. 1E) and to keep the lead outside of the cell together with other compounds in the CW (Fig. 2B, F, J, N). Other microorganisms with mutated gene transport system called fast and unspecific were reported to have mutants tolerant of metals. For example, Cor A mutants were tolerant of Co (II; Bui *et al*., 1999; Pfeiffer *et al*., 2002) and Pit mutants were tolerant of arsenate (Rosen, 1999). Data show that *Candida* species may have a mutation in the lead transportation system, which confers resistance to this metal. Another mechanism by which *Candida* species can maintain extracellularly lead may result from carriers that are in the membrane contributing to their resistance to this metal. So far, in *Candida* species, there have not been reported any facilitators of this cation that allows efflux. However, lead transporters can be the ATP‐dependent transporters (ABC) and facilitators (MDR), known as CDR and MDR, respectively, which have been reported as protein transporters for multiple drugs (Ramage *et al*., 2002; Mukherjee *et al*., 2003; Anderson, 2005; Cuellar‐Cruz *et al*., 2012). These transporters may be responsible for lead efflux in *Candida,* as reported in bacteria. The most studied ATPase type P is the protein CadA encoded in plasmid *p*I258 from *Staphylococcus aureus* (Yoon and Silver, 1991). In the genome of *Cupriavidus metallidurans* strain CH34, the presence of genes encoding ATPases type P is notable, which are collectively involved in the homeostasis or in the resistance to cadmium, copper, lead and zinc (Nies, 2003). The formation of extracellular MCs by the four species of *Candida* is the first evidence that these pathogenic fungi have been exposed to environments with high concentrations of lead, such as the bloodstream or organs of the host. The second evidence is their adaptability to this hostile environment in order to survive. These data open the possibility of studying and determining not just the mechanisms of assimilation but also the resistance of *Candida* to heavy metals, therefore enlightening our understanding of the pathogenesis and virulence of these yeasts in human host. In the case of Hg^2+^, *C. albicans*,* C. glabrata* and *C. krusei* form mainly intracellular MCs (Fig. 2C, G, K), in contrast to *C. parapsilosis* that has not been observed to form MCs, although the cells show apparent structural damage (Fig. 2O). However, in the presence of Cd^2+^, four species of *Candida* were found to form intracellular NCs (Fig. 2D, H, L, P). These intracellular NCs were observed like lights inside the yeasts (Fig. 2). Unlike lead, the formation of intracellular MCs or NCs from mercury or cadmium, respectively, was probably due to the fact that *Candida* species are not normally exposed to these metals. This is why their mechanisms have not resistance to enable them to maintain these extracellular cations. Even though *Candida* is unable to replicate in the presence of Hg^2+^ or Cd^2+^ (Fig. 1F, G), it can, nonetheless, form MCs and NCs (Fig. 2C, D, G, H, K, L, P) because its metabolism appears to be mainly directed to synthesizing MCs or NCs. As a first step in the formation of intracellular MCs or NCs, *Candida* needs to import metals to the cytoplasm through any of the systems that capture heavy metal cations (Nies and Silver, 1995). Once inside the cell, the excess of metals can form coordinate bonds with anions that block functional groups of enzymes to inhibit the transport system by moving essential metals from native binding sites and by disrupting the integrity of the cell membrane (Nies, 2003). Thus, the facility of Cd^2+^ to move to the cytoplasm enables the interaction with the membrane transporters involved in the capture of Zn^2+^, Ca^2+^ or Fe^2+^, displacing these metals (Bridges and Zalups, 2005). Therefore, cadmium will be able to replace these cations and form complex coordinate covalent bonds with biomolecules and sulfhydryl groups. This may result either in the inhibition of the activity of sensitive enzymes or in the reaction with molecular oxygen (Kachur *et al.,* 1998). Calcium channels provide one of the main entrances of Cd^2+^ to the cell, considering that Cd^2+^ and Ca^2+^ have similar ionic radius (Goyer, 2003; Mendez‐Armenta and Rios, 2007; Flora *et al*., 2008).

The alteration in the homeostasis of intracellular calcium leads the cell to release mitochondrial and endoplasmic reticulum calcium, thereby producing alterations in the metabolism. Hg^2+^ can enter the cytoplasm and be methylated by the microorganism, forming methyl mercury (Pan‐Hou and Imura, 1982). This methyl mercury complex is structurally similar to methionine, so that its transport into the cell is possible through the protein transporter of neutral amino acids. It has been reported that when the cell gradually accumulates metal cations, there tends to be a homeostasis of heavy metals within the cell. Thus, the cell adapts by counteracting the effects of high concentrations of metal ions (Trajanovska *et al*., 1997). This process (a type of homeostasis and adaptation of *Candida* species to heavy metals) is directly related to the formation of intracellular MCs and NCs. In the biosorption of Cd^2+^ or Hg^2+^, the *Candida* CW plays an important role as it facilitates the entry of these cations. Studies have also reported that, at a pH between four and six, the negative charge of the fungal wall favours ionic approach with metal ions so these will enter the cell (Loukidou *et al*., 2004). Data show that in *Candida* species, the pH of the CW changes depending on the metal that is present. The fact that *C. parapsilosis* cannot form MCs in the presence of Hg^2+^ may be due, in part, to the composition of its CW, which differs from that of *C. albicans, C. glabrata* and *C. krusei* (Silva *et al*., 2012). The apparent damage to the CW may be due to mercury generating high concentrations of OS and ROS. Previous studies show that OS generates damage to the CW of *Candida* species (Ramirez‐Quijas *et al*., 2015). Moreover, the exporter proteins of *Candida species* are specific for certain cations; subsequently, lead (Pb^2+^) will not be able to bind to these systems.

**Figure 3:**
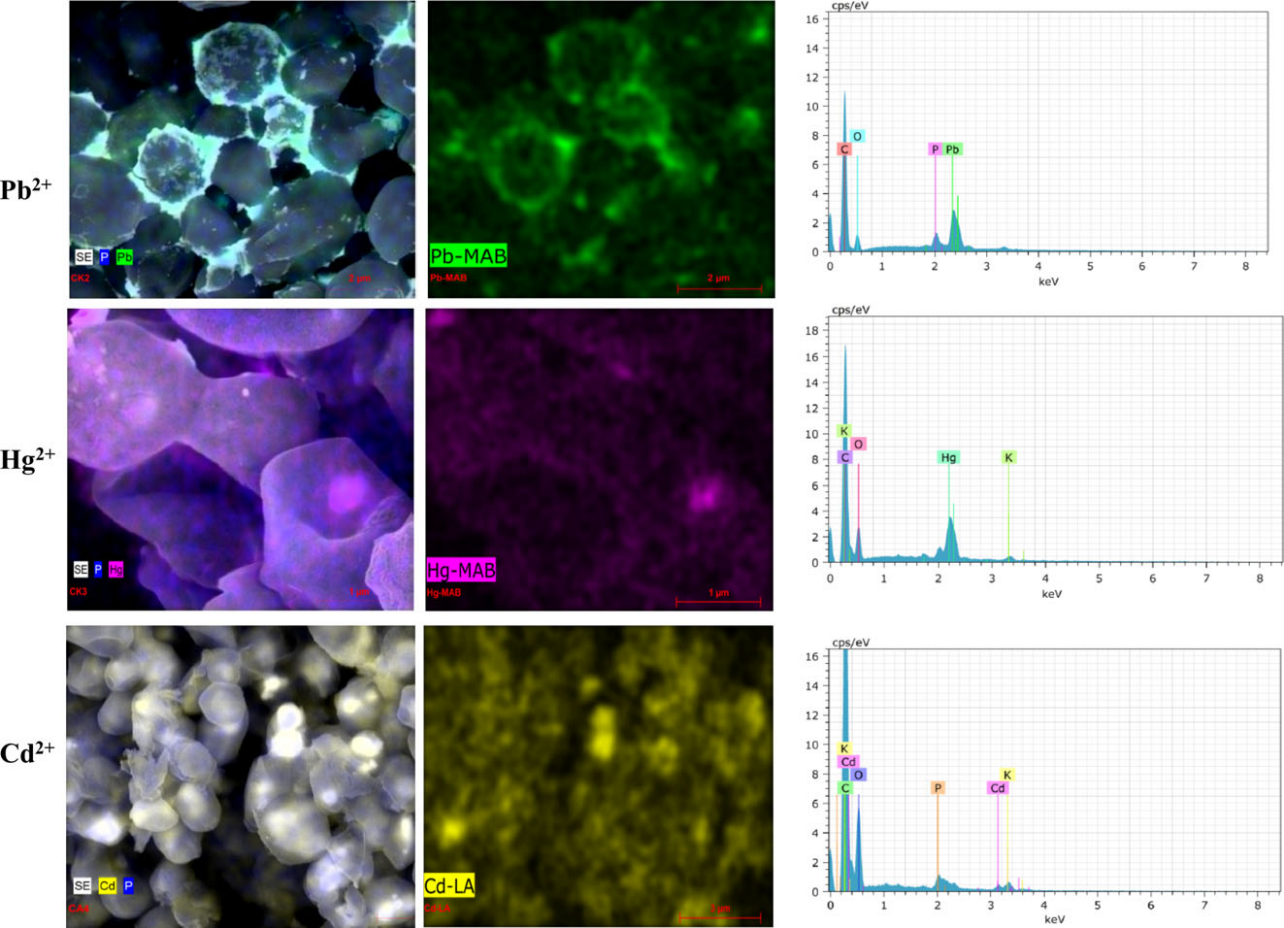
Qualitative analysis of elements present in the micro‐ or nanocrystals by energy‐dispersive spectroscopy (EDS). The samples of *Candida* species were observed by SEM and were analysed qualitatively in order to determine their main components. As shown, micro‐ or nanocrystals are formed depending on the metal that they were exposed.

We have additionally performed a qualitative analysis of the elements present using energy‐dispersive spectroscopy (EDS) in order to confirm that the MCs and NCs observed by SEM were indeed MCs or NCs of Pb^2+^, Hg^2+^ or Cd^2+^ sulfides. The representative EDS signal in *C. albicans* and *C. krusei* (in the analysed MCs and NCs) was found either for lead, or for mercury or for cadmium as appropriate in both the extracellular and intracellular MCs and NCs (Fig. 3). These results show that *Candida* species have specific input mechanisms and resistance to Pb^2+^, Cd^2+^ or Hg^2+^. This suggests a typical biomineralization process (biogenic crystallization) that achieves homeostasis at a high concentration of these heavy metals.

### Tailored synthesis of PbS, HgS, HgCl_2_ and CdS crystals by *Candida* species

**Figure 4:**
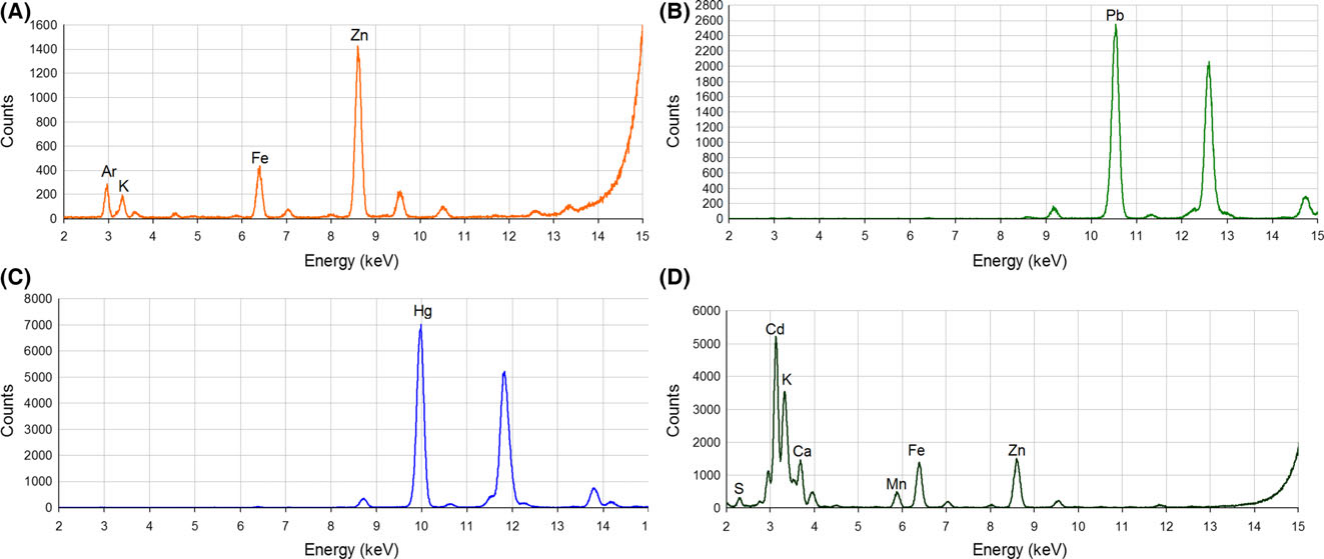
Major fluorescence peaks interpretation for (A) *Candida* blank samples packed in capillary tubes – elements labelled on corresponding K_α_ lines. B. Peaks for a *Candida* lead‐loaded samples belong to Pb; each element is labelled on its L_α_ lines. C. Peaks for a *Candida* mercury‐loaded sample belong to Hg; each element is labelled on its L_α_ lines. D. Peaks interpretation for a *Candida* cadmium – elements are labelled on corresponding K_α_ or L_α_ lines.

The ability to form MCs or NCs is apparently specific to some species of *Candida*. SEM showed that some species of *Candida* such as *C. albicans* and *C. glabrata* have higher affinity for metals than for others. It is this higher affinity for metal that enables them to form MCs or NCs (Figs 2 and 3). In order to elucidate the chemical composition of the MCs and NCs formed by each of the *Candida* species for each individual cation, MCs and NCs were analysed through synchrotron radiation XRD‐XRF. Synchrotron radiation is by now the only tool to determine the chemical composition of low amount samples, low concentrations and also to analyse crystals of the size obtained in this study. Usually, the home powder X‐ray diffractometers do not have the appropriate sample holders for a limited proportion of mass of these samples. The fluorescence spectra from synchrotron radiation confirmed the presence of expected bioaccumulated heavy metals in lyophilized cells, with superimposable patterns among different cell lines (Fig. 4, Table S2). Furthermore, endogenous zinc and other common metals (K, Fe) have been found, even in blank samples (Fig. 4A).

**Figure 5:**
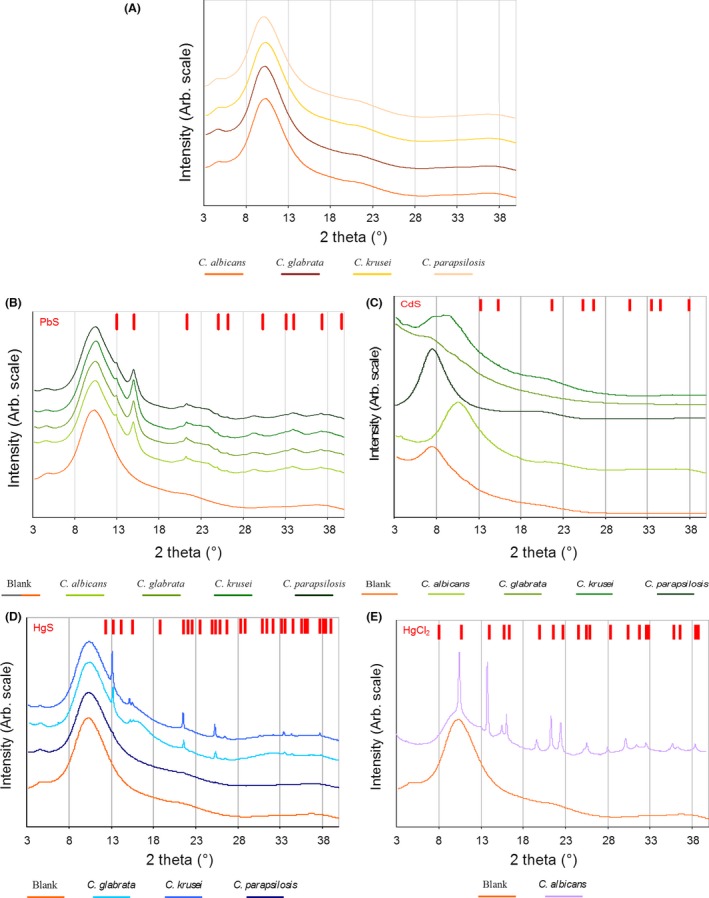
X‐ray powder patterns collected on *Candida* cells. A. Cells not exposed to heavy metals (controls). B. *Candida* cells exposed to Pb^2+^. Red bars represent expected positions of cubic $Fm\overset{¯}{3}m$ PbS. C. *Candida* cells exposed to Cd^2+^. Red bars represent expected positions of cubic $F\overset{¯}{4}3m$ CdS. D. *C. glabrata*,* C. krusei* or *C. parapsilosis* cells exposed to Hg^2+^. Red bars represent expected positions of trigonal *P*3_2_21 HgS (Auvray and Genet, 1973). E. *C. albicans* cells exposed to Hg^2+^. Red bars represent expected positions of tetragonal *I*4*/mmm* HgCl_2_ (Adams *et al*., 1970; Boggon and Shapiro, 2000). Patterns vertically shifted for clarity.

X‐ray powder patterns collected from *Candida* cells, not exposed to heavy metals (blanks), showed broad peaks that are in agreement with previous data published for glucans extracted from CW (Lowman *et al*., 2014) and ‘poorly ordered’ lipid phases (Fidan *et al*., 2014; giving the broad peak at ~4.3 Å). Blank patterns of the four *Candida* species analysed are plotted and compared properly (Fig. 5A).

The presence of heavy atoms (which belong to crystalline metallic micro‐ or nanoparticles) in the cells added more sharp signals to the blank background. For lead‐loaded cells (Fig. 5B), the sharp signals nicely matched the peak positions expected for crystalline cubic $Fm\overset{¯}{3}m$ galena (PbS) phase (Noda *et al*., 1987) samples from different cell lines that were equivalent. These results show that MCs which appeared in the presence of Pb^2+^ in the four species of *Candida* were formed by PbS (Figs 4B and 5B). Galena (PbS), lead sulfide with halite‐type structure, is the most important lead mineral in the Earth's crust. PbS is further interesting because it is a naturally occurring semiconductor. The atomic arrangement of galena is the same as that of NaCl, that is cubic closed‐packed, with Pb atoms in the octahedral interstices. If a Born ionic model is assumed for galena, periodic bond chain (PBC) analysis of crystal morphology (phase) gives similar results as those for the NaCl case.

The mechanism by which *Candida* species promote the synthesis of PbS MCs extracellularly involves the CW in the formation of covalent coordinate bonds between the metal ion with other components of the CW, favouring an acid pH. This process depends on the degree of protonation of the CW (Gupta *et al*., 2000). The photomicrograph of the SEM shows that *C. albicans* cells are found only as yeast form and not as hyphae in acidic pH (Fig. 2B). However, under extracellular alkaline pH, they will have a hyphal growth as shown *in vitro* (Davis *et al*., 2003). Furthermore, it has been reported elsewhere that the ability of *C. albicans* to respond to changes in extracellular pH is controlled, in part, by changes in the gene expression. The *PHR2* gene encodes for a CW protein involved in binding of β‐1,3 and β‐1,6 glucans expressed in acidic conditions (Muhlschlegel and Fonzi, 1997). In addition, transcriptional analyses in *C. albicans* have shown that in response to the changes in extracellular pH, nearly five hundred genes are regulated, either by alkalization or by acidification of the medium. Of these, 267 genes are activated in response to pH and, among these, a significant number are related to iron metabolism (Bensen *et al*., 2004). Some of the genes involved in iron metabolism may also be involved in the metabolism of lead, which could partly promote the synthesis of PbS MCs in the CW of *Candida* species. Additionally, *Candida* species ensure an extracellular acidic environment in the presence of Pb^2+^ and ATPase of the membrane Pma1. A similar mechanism in *S. cerevisiae* has also been reported to have proton export activity (Vanderrest *et al*., 1995).

On the other hand, synchrotron radiation analysis of the MCs formed in the presence of Hg^2+^ showed different chemical composition for each of the *Candida* species. The diffraction patterns also showed significant differences for mercury. Among the different samples (Fig. 5D, E), XRD peaks for *C. glabrata*,* C. krusei* and *C. parapsilosis* can be interpreted as cinnabar (HgS) trigonal *P*3_2_21 crystals (Auvray and Genet, 1973; Clever *et al*., 1985). It should also be noticed that *C. parapsilosis* signals are much weaker compared with other cell lines (Fig. 5D). This suggests that mercury is less efficient for *in vivo* crystallization. This result is in agreement with that found in the SEM, where no MCs of mercury were observed for this particular *Candida species* (Fig. 2O). Mercury‐loaded *C. albicans* shows a completely different powder profile, suggesting the presence of calomel particles (HgCl_2_, with a tetragonal *I*4*/mmm* space group; Barnes and Bosch, 2006; see Fig. 5E).

The synthesis of the HgS MCs formation of *C. glabrata, C. krusei* and *C. parapsilosis* is performed as described in other microorganisms (Wood *et al*., 1968; Landner, 1972; Bisogni and Lawrence, 1973). The biomineralization of HgS MCs in *C. glabrata, C. krusei* and *C. parapsilosis* requires an acidic pH, which can be maintained in an intracellular manner through any of the mechanisms described in other fungi. One such mechanism is through the Nha1 antiporter, which in *S. cerevisiae* has the principal function of continuous recycling of potassium cations through the plasma membrane, in homeostasis of intracellular K^+^ and at a specific pH value. Its contribution to the detoxification of Na^+^ and Li^+^ ions is important, but not crucial. Another antiporter involved in intracellular pH is Kha1, which is an antiporter of Na^+^/H^+^ and is located in the membrane of the Golgi apparatus. As in the case of other intracellular transporters, cations of alkali/H^+^ metal yeast, it is involved in regulation of potassium within the organelle and pH homeostasis. Nha1 and Kha1 (together with the vacuolar H^+^‐ATPase) are involved in the intraorganellar pH and in the balanced alkali metal cations (Arino *et al*., 2010; Cyert and Philpott, 2013).

In the particular case of HgCl_2_ MCs formed by *C. albicans*, mercuric ions are biomineralized in a different mechanism. This difference in the way of mineralizing certain heavy metals is due to *C. albicans* that instead of taking S^2−^ ions uses the anion Cl^−^ to detoxify Hg^2+^. The existence of various mechanisms that these yeasts have developed to deal with toxic metals indicates that they have been subjected to different niches.

Synchrotron radiation diffraction, on cadmium‐loaded cells, has been measured on small quantities of lyophilized cells. The high toxicity of this metal for *Candida*, which inhibited cell proliferation (Fig. 1G), suggests the absence of efficient detoxification mechanisms for this metal in *Candida*. Furthermore, the bioaccumulated metal gives very small diffraction peaks, which could be explained by the presence of tiny (‘quantum’) crystalline particles, as previously reported in the literature (Williams *et al*., 1996). X‐ray diffraction peaks are more pronounced in the *C. glabrata* pattern and their positions correspond to the expected positions of peaks for a cubic $F\overset{¯}{4}3m$ Hawleyite (CdS) phase (Malik *et al*., 1993; Barnes and Bosch, 2006; Shakouri‐Arani and Salavati‐Niasari, 2014; Fig. 5C). The cubic crystals of Hawleyite (CdS) usually show halite‐type structure as that found for galena previously mentioned. Hawleyite is a rare sulfide and usually appears as a bright yellow coating on sphalerite; it is usually precipitated and confused with the mineral greenockite, which crystallizes in a hexagonal group and appears as an orange‐yellow colour. Slight shifts in the angular position could suggest differences in lattice parameters of CdS, which may be due to crystallization of Cd‐Zn sulfide solid mixtures (Noor *et al*., 2010). The formation of CdS NCs has already been reported for *C. glabrata* and *S. pombe* (Dameron *et al*., 1989). The intracellular formation mechanism is performed when Cd^2+^ ions in the cytoplasm are complexed with γ‐Glu peptides (Grill *et al*., 1986; Hayashi *et al*., 1986). These complex metal–γ‐peptides incorporate sulfide ions arising from a Cd‐mediated enhancement of cellular sulfide generation (Murasugi *et al*., 1984; Mehra *et al*., 1988; Reese and Winge, 1988). In the formation of HgS and HgCl_2_ MCs or CdS NCs, the process requires an acidic intracellular pH, which *Candida* maintains in the previously described mechanisms.

**Figure 6:**
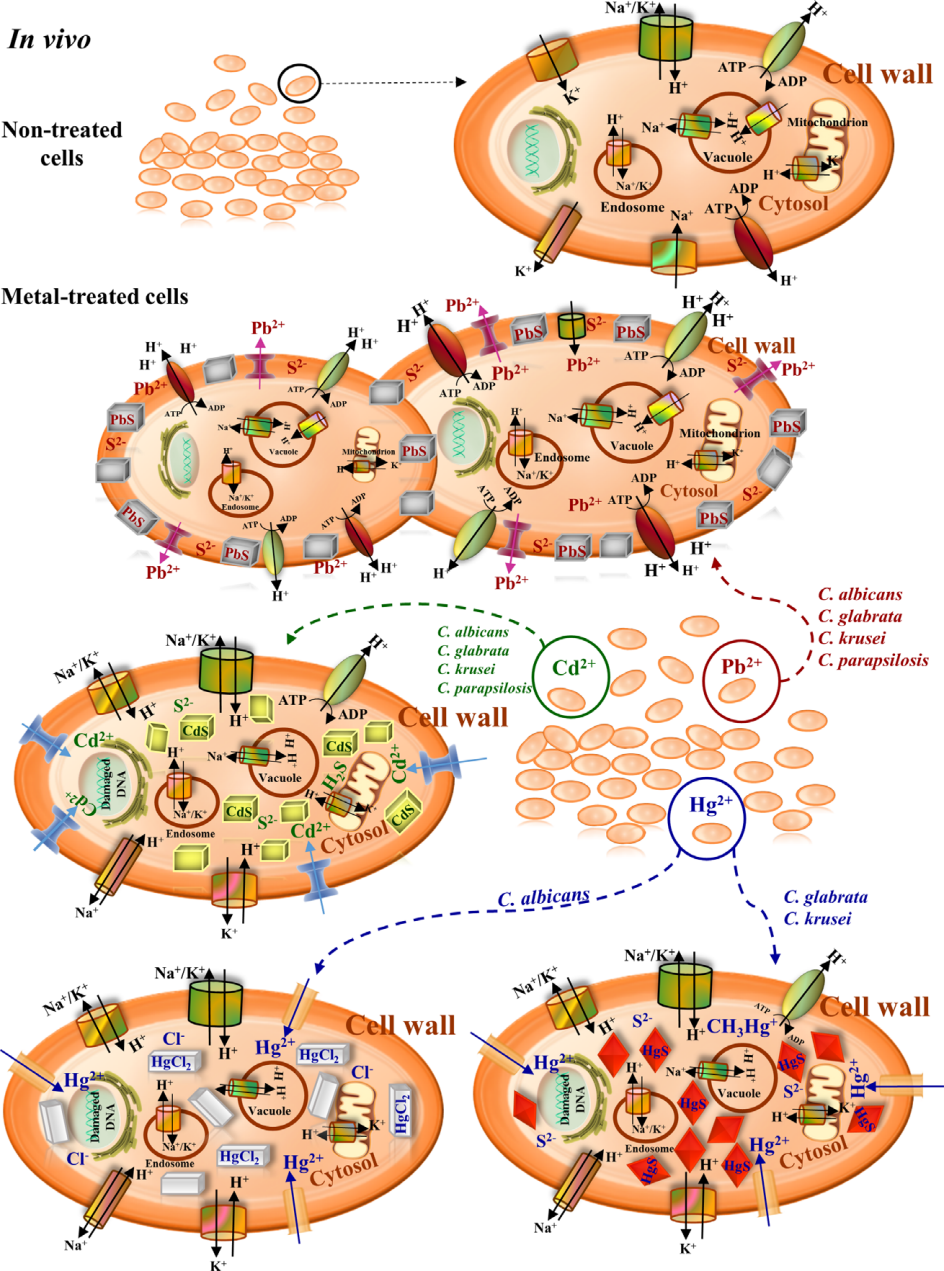
Proposed mechanism by which *Candida* species synthesize micro‐ or nanocrystals from PbS, HgS, HgCl_2_ or CdS. Control cells are both metabolized and replicated. Pb^2+^‐treated cells are able to replicate and to detoxify metal‐forming extracellular PbS MCs. Although cells treated with either Hg^2+^ or Cd^2+^ failed to replicate, they achieved homeostasis by intracellular formation of MCs or NCs from HgS, HgCl_2_ or CdS respectively. In the presence of mercury salts, *C. albicans* used to form MCs of HgCl_2_; though, *C. glabrata* and *C. krusei* synthesized MCs of HgS. *C. parapsilosis* was not able to synthesize MCs of mercury. In the formation of both extracellular and intracellular MCs and NCs, cells of *Candida* need to maintain an acidic pH value either intracellular or extracellular.

These results show that each species of *Candida* has developed specific and differential mechanisms to detoxify each of the metals to which they have been subjected in the various environmental and physiological habitats. The mineralization *in vivo* is one of the principal mechanisms by which cell homeostasis, with the cation, occurs through the formation of MCs and NCs (Fig. 6). Thus, in conclusion, biomineralization through crystallization is a fundamental chemical mechanism produced from microorganisms to higher organisms in order to perform basic functions, and it can be used as a defence mechanism against toxic elements, such as that produced by *Candida* species that form micro‐ or nanocrystals to reach homeostasis in the presence of toxic metals.

In order to complement the *in vivo* experiments, we decided to synthesize sulfides of different metals (Pb^2+^, Hg^2+^ and Cd^2+^) *in vitro*. We based our synthesis strategy in the cell growth information where four types of *Candida* species were exposed to these heavy metals. The *in vitro* experiments showed that the synthesis of the PbS, HgS and CdS is feasible when the pH of the reaction is acidic through diffusing‐reacting systems, either agarose gel or those gels obtained from the neutralization of sodium metasilicate.

### Synthesis of XS (X: Pb^2+^, Hg^2+^ and Cd^2+^) and crystal growth experiments

Years of experimenting with different crystals has confirmed that by minimizing the convective transport of mass, we will generally obtain higher‐quality crystals, with improved mechanical and optical properties, reduced density of defects and of larger size. There are two approaches that suppress or at least reduce the convection in crystal growth that demonstrated this assumption. One is the crystallization in space (Littke and John, 1984; Long *et al*., 1994; Kundrot *et al*., 2001), and the other is the crystal growth in gels, which produces crystals of high quality for high‐resolution X‐ray crystallography compared with those crystals obtained in solution (Lorber *et al*., 2009). The way of reducing the natural convection of solutions under Earth gravity is by incorporating jellified media into the solutions (Garciaruiz, 1991). Although the gel growth method is known since the end of the nineteenth century from the experiments performed by Liesegang (1896) about periodic precipitation (cited elsewhere; Henisch and Garciaruiz, 1986a,b), it has not been sufficiently explored in the growth of inorganic/organic crystals (Robert and Lefaucheux, 1988).

**Figure 7:**
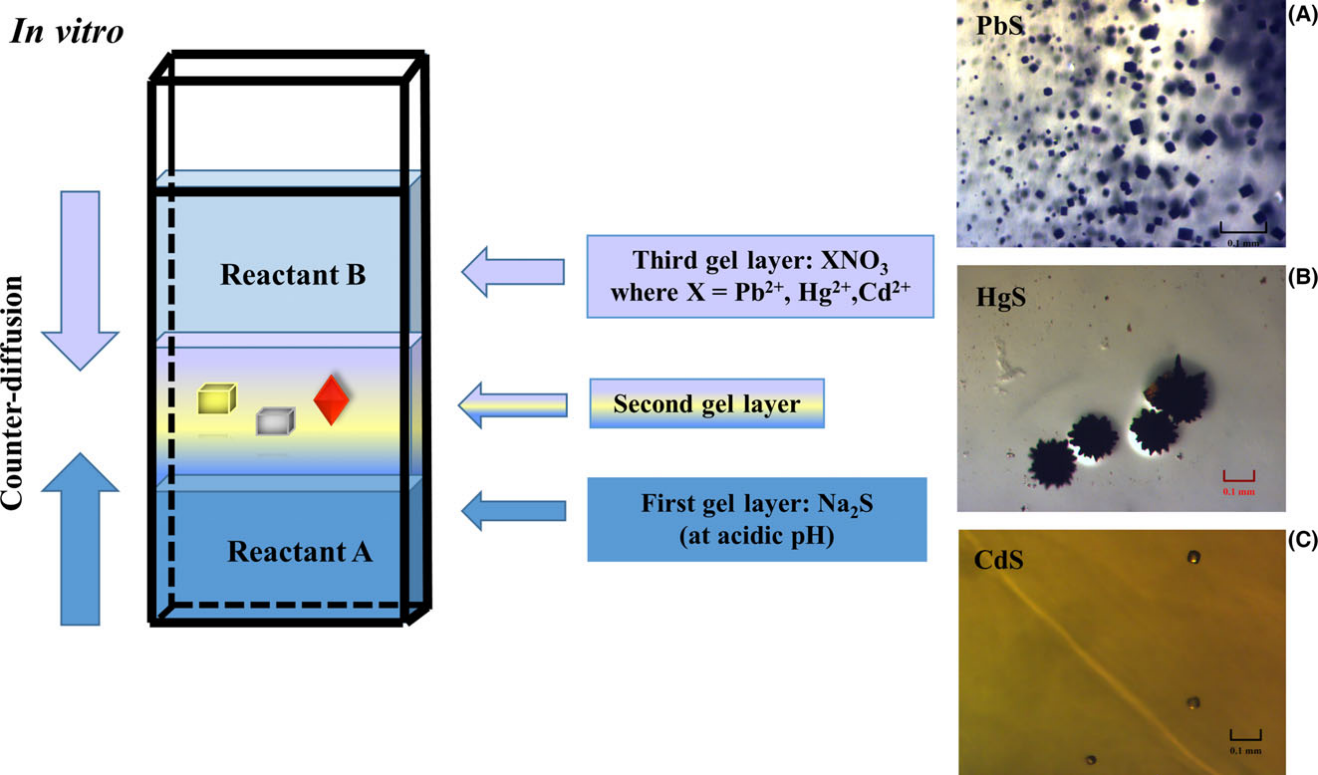
Crystallization of XS (X:Pb (II), Hg (II) and Cd (II)) in hydrogels. The figure on the left shows the experimental set‐up used for growing these *in vitro*‐grown crystals. The sandwich gel part is the place where all crystals are obtained by the counter‐diffusion of all the ions. The right image corresponds to the synthetically obtained crystals of these three sulfides: (A) the upper part shows the grey metallic cubic‐shaped PbS crystals, (B) HgS a red‐dark spherulites and (C) tetragonal‐shaped crystals of CdS at the end of the sandwich layer of the gel.

For the *in vitro* synthesi*s* of sulfides of heavy metals, a Granada Crystallization Box (Garcia‐Ruiz *et al*., 2002) from Triana Sci and Tech (GCB, Spain) was used by means of the gel growth method in three‐layer configuration (Fig. 7). The first layer of the gel contained one of the components, which was Na_2_S (reactant A) of the synthesis after which a sandwich layer was prepared (without any reactants). The second component (reactant B) of the reaction was poured onto the top of the sandwich gel (third gel layer). The components of the reaction counter‐diffused each other to react in the central part of the sandwich gel, which was the second gel layer without any of the reactants of the reaction. MCs of PbS, HgS and CdS were synthesized according to the reactions described in the paragraphs below (these sulfides are virtually insoluble in water). The *in vitro* experiments showed that the synthesis of the PbS, HgS and CdS is feasible when the pH of the reaction is acidic through diffusing‐reacting systems, either agarose gel or those gels obtained from the neutralization of sodium metasilicate.

The reactant A (0.1 M Na_2_S initially prepared in an acidic buffered solution using phosphoric acid at pH 2) was introduced to the first gel layer mixing 1:1 with 1% (w/v) agarose. The reactant B (each of the aqueous solutions of XNO_3_ salts, where X: Pb^2+^, Hg^2+^ and Cd^2+^) was incorporated to the third gel layer following the same procedure. For crystals of galena (PbS) grown in gels, harvested crystals were evaluated by powder X‐ray diffraction and EDS‐SEM. These microcrystals matched the peak positions expected for crystalline cubic $Fm\overset{¯}{3}m$ of galena phase in X‐ray diffraction and characteristic peaks for the metal and sulfur in EDS‐SEM. These crystals showed a classical cubic‐shaped habitats and a grey metallic colour (Fig. 7A) as reported elsewhere (Garciaruiz, 1986; Noda *et al*., 1987). These galena crystals grown *in vitro* showed a characteristic crystal growth pathway: first, they started to grow a dendritic hollow shape, followed by a quasi‐cubic shape (when the dendrite was completely filled) up to a perfect cubic shape (Garciaruiz, 1986). They finally reached a classical cubic shape at the end of the sandwich gel layer, and compared with the other sulfides, these galena crystals were well‐formed cubes.

Though, we followed the same method of crystal growth for synthetic crystals of cinnabar (α‐HgS, dark‐red colour mixed with black powders (β‐HgS), appear at the beginning of the crystallization reaction. These cinnabar (α‐HgS) red crystals were characterized by powder X‐ray diffraction showing a trigonal *P*3_2_21 space group (Auvray and Genet, 1973), whereas EDS‐SEM showed the characteristic peaks for Hg and sulfur. In the lower part of the sandwich gel layer, red tiny spheres at the beginning up to dendrites of this mercury (II) sulfide were observed (Fig. 7B).

Finally, crystals of cadmium (II) sulfide were obtained in a fine yellow powder at the beginning of the reaction in the sandwich gel layer. When both concentrations were counter‐diffused and properly equilibrated, tiny octahedral‐shaped crystals were obtained (Fig. 7C). The expected positions of peaks from powder X‐ray diffraction corresponded to characteristic diffractions for a cubic $F\overset{¯}{4}3m$ Hawleyite phase (Shakouri‐Arani and Salavati‐Niasari, 2014) and EDS‐SEM showed the characteristic peaks for cadmium and sulfur.

### MCs and NCs synthesized *in vivo* and *in vitro* exhibit the same crystalline morphology

**Figure 8:**
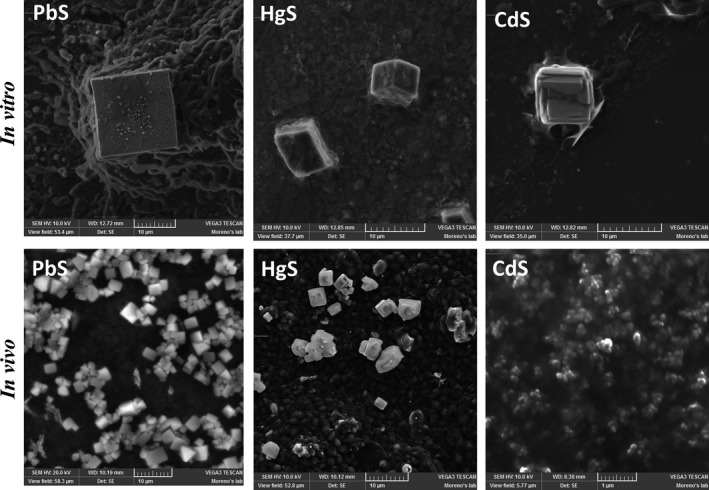
Comparison of heavy metals sulfides. The *in vitro* crystals in the upper part show the SEM micrographs of these sulfides of Pb (II), Hg (II) and Cd (II) obtained in hydrogels respectively. The lower part of the figure shows the most representative microcrystals of PbS and HgS and nanocrystals of CdS obtained in these four species of *Candida*.

As illustrated in Figs 7 and 8, MCs of PbS and HgS synthesized by *Candida* cells show the same crystalline morphology and shapes than those obtained *in vitro*. However, those formed *in vivo* produced higher amounts, but different in size. These characteristics make *Candida* cells useful producers of PbS and HgS, which can be utilized in a number of different technological areas as described previously (Williams *et al*., 1996; Kowshik *et al*., 2002; Krumov *et al*., 2007; Korbekandi *et al*., 2014). Interestingly, *Candida* also synthesizes CdS crystals that look like clusters in the form of bunches (Fig. 8). They appear in the form of lights inside the cell when observed by SEM (Fig. 2). CdS crystals obtained in *Candida* species are produced in considerable amounts although of smaller size. However, they exhibit the same crystalline morphology as those obtained *in vitro*. As we described along this contribution, PbS, HgS and CdS can be technologically important materials. All these results indicate that *Candida* yeasts can not only produce higher amounts but also more stable MCs and NCs than those formed *in vitro*. All these properties make *Candida* a potential alternative in future biotechnological developments and applications.

## Conclusions

This contribution showed how *Candida* species have a singular way of synthesizing either sulfides or chlorides of different heavy metals. Our *in vivo* results showed that in the presence of Pb^2+^, *Candida* cells are able to replicate and form extracellular PbS MCs, whereas in the presence of Hg^2+^ and Cd^2+^, the cells fail to replicate. However, they did synthesize intercellular MCs or NCs from HgS or HgCl_2_ and CdS respectively. This shows that these *Candida* species adapt differentially to the environment, counteracting the effect of the heavy metals. The synthesis of MCs and NCs is a form of homeostasis and adaptation of these fungi. These crystals grown *in vitro* were bigger in size (in some cases) than those observed in *Candida*. However, the synthesis in gels is anything but simple. This is due to the number of chemicals used to produce the hydrogel as well as for the reactants used to complete this synthetic reaction. On the other hand, in *Candida*, there are plenty of biological machineries that are activated by genes in the synthesis of these micro‐ or nanocrystals of heavy metal sulfides. We can finally infer from these results that in the near future, these *Candida* species could be used either as biologically based decontaminants or as specific heavy metals removal.

## Experimental procedures

### Strains and culture conditions

The strains of *C. albicans*,* C. glabrata*,* C. krusei* and *C. parapsilosis* used in this study are clinical isolates from blood cultures of the collection of Department of Microbiology, ENCB‐IPN, México. Yeast strains were cultured on yeast peptone (YP; yeast extract, 1%; peptone, 2% glucose), and 2% agar was added to solidify the media (Ausubel *et al*., 2001). The formation of MCs and NCs was induced by the addition of lead nitrate, mercury nitrate or cadmium nitrate (all obtained from Sigma‐Aldrich Toluca, Mexico) to the culture medium, prior to yeast inoculation.

### Susceptibility testing of Pb(II), Hg(II) or Cd(II)

Cells in the exponential phase of each *Candida* species at OD_600nm_ 1.0 were divided and exposed to different concentrations (0, 0.5, 1.0, 1.5 or 2.0 mM) of each of the heavy metals Pb^2+^, Hg^2+^ or Cd^2+^. The next step was to incubate them with shaking at 28°C for 48 h to allow the cells to reach the stationary phase. Subsequently, aliquots of the control and treated cultures with different metals were taken and adjusted to an OD_600 nm_ 0.5. With these aliquots, exponential dilutions were made in 96 well boxes and each dilution was spotted on to YPD plates and incubated at 28°C for 48 h (Cuellar‐Cruz *et al*., 2008). The plates were photographed in the plate reader Gene genius bioimaging system, Syngene. The experiments were performed in duplicate.

### Growth curve

A preinoculum of each of the four species of *Candida* was added to 25 ml of YPD liquid medium and incubated at 28°C with constant stirring. From this culture every 2 h for 48 h, the OD_600 nm_ was measured using a Genesys 20 (Thermo Scientific) spectrophotometer. The cells exposed to heavy metals (Pb^2+^, Cd^2+^ or Hg^2+^) had a final concentration of 1.0 mM of the corresponding compound. Finally, the OD_600 nm_ was plotted against time in hours (h). Two independent experiments were performed.

### Isolation of MCs and NCs after lysis of *Candida* protoplasts

To isolate MCs and NCs, yeast protoplasts were obtained as follows: cells of the four *Candida* species treated with Pb^2+^, Hg^2+^ or Cd^2+^ were pelleted by centrifugation at 3500 × g for 15 min at 4°C, and the pellets were washed four times with sterile deionized water, resuspended in water and counted. Aliquots of the cell suspension were resuspended at a final OD_600 nm_ of 1.0 in 1.0 ml of lysis buffer containing 50 mM Tris–HCl, pH 7.2, 0.8 M sorbitol, 0.8 M KCl, 10 mM MgSO_4_, 15 mM β‐mercaptoethanol and 0.25 mg ml^−1^ lyticase (all reagents from Sigma‐Aldrich) and incubated at 37°C. After 3 h, cells were observed with an Zeiss Axiostar microscope (Carl Zeiss, Jena, Germany) to assess protoplast formation. This was about 90%. Protoplasts were collected and gently lysed by resuspending those in 500 *μ*l of sterile deionized water, and MCs or NCs formed *in vivo* were pelleted and separated from cellular debris by centrifugation at 120 × *g* for 3 min.

### Scanning electron microscopy (SEM)

The cells of *C. albicans, C. glabrata, C. krusei* and *C. parapsilosis*, after exposure to Pb^2+^, Hg^2+^ or Cd^2+^, were centrifuged at 3500 × *g* for 10 min at 4°C, and cell pellet was thoroughly washed four times with sterile deionized water. The cells were then lyophilized in a Tousimis Auto‐Samdri critical point dryer 815 for 4 h. The dried samples were covered with a layer of colloidal gold. Then, the samples were observed in the scanning electron microscope, model EVO HD15, high‐definition ZEISS^®^. Finally, the samples were photographed using the secondary electron detector (SE1) at 15 kV in high vacuum conditions at a working distance of 4 mm. We used, additionally, a SEM from TESCAN (Brno, Czech Republic) model VEGA3 SB, for obtaining the images of the crystals synthesized *in vitro* through a secondary electron detector (SE) from 10 to 20 kV in high vacuum conditions (work distance of 10 mm). All crystalline samples were sputtered with a gold film in order to increase the resolution imaging and contrast.

### X‐ray powder diffraction (XRPD)

X‐ray powder diffraction (XRPD) analysis was performed at the X‐ray diffraction beamline (XRD1) of the Elettra Synchrotron, Trieste (Italy; Lausi *et al*., 2015). Powder diffraction patterns were collected, in transmission mode, at room temperature (25°C) with a monochromatic wavelength of 0.77491 Å (16 keV) and 200 × 200 *μ*m^2^ spot size, using a Pilatus 2M hybrid‐pixel area detector. Samples of lyophilized *Candida* cells were packed in borosilicate capillaries (700 *μ*m diameter and 10 *μ*m wall thickness). Blank samples were analysed in the same way, collecting data on lyophilized cells not exposed to heavy metals, but treated with the same protocol (growth parameters, washing and lyophilization steps). The small amount of cadmium‐loaded samples available (limited by high metal toxicity) prevented the usage of capillaries; these powders were therefore ‘glued’ on a 300 *μ*m circular Kapton loop using N‐paratone. Blank pattern for these samples refers to diffraction of the empty loop containing similar amount of oil. Bidimensional powder patterns have been integrated using Fit2D program (Hammersley *et al*., 1996; Hammersley, 2016), after preliminary calibration of hardware set‐up, using a capillary filled with boron lanthanide (LaB_6_) standard reference powder (NIST 660a). Fluorescence spectra have been recorded for all the samples, during diffraction data acquisition on a Silicon drift Amptek X‐123SDD detector, perpendicular to X‐ray beam.

### Synthesis of crystals *in vitro* and crystal growth in different kinds of gels

Crystals of PbS, HgS and CdS were synthesized by the gel method using two types of hydrogels: the first one was a low melting point agarose, and the second one was a gel obtained by the neutralization of sodium metasilicate solution with acetic acid. In the following paragraphs, we describe each of the gel preparations and recipes used in the chemical synthesis of these crystals:

#### Agarose preparation

Agarose gel 1.0% (w/v) stock solution of low melting point agarose (T_gel_ = 20°C, Hampton Research Cod. HR8‐092) can be prepared following the conventional procedure: dissolve 0.1 g agarose in 10 ml of water heated at 90°C until obtaining a transparent solution when stirring. Then, this solution is passed through a 0.22 *μ*m porosity membrane filter for removing all dust particles or insoluble fibres of agarose. The gel solution is kept at 10°C in the fridge in order to avoid any contamination. Prior to the crystallization in agarose, the gel can be heated at 90°C by using a heating plate in order to melt the agarose. The mixture 1:1 of 1% (w/v) agarose and 0.1 M Na_2_S (prepared in buffer phosphate pH 2.0) is obtained. This helped us to obtain the first gel layer with one of the reactants included in the agarose (reactant A). Then, a second layer of 0.5% (w/v) agarose in water (ready for crystal growth experiments) is poured onto the top the first gel layer. It is important to emphasize that this sandwich gel layer must not contain any of the two reactants of the precipitating reaction. The second reactant (called B and located in the third gel layer, which is a jellified solution of the heavy metal nitrate (XNO_3_: X can be Pb^2+^, Hg^2+^ and Cd^2+^)) is poured onto the top of the second gel layer. The sulfur ions counter‐diffuse through the second layer of the gel. The second reactant (XNO_3_) of the reaction is also counter‐diffused through the second layer to produce the crystals in the middle of the sandwich gel layer. Although, generally speaking, agarose has been the most popular gel for the crystallization of different substances, there are other types of gels that have also been used for the crystallization of organic/organometallic substances when organic solvents are needed (Choquesillo‐Lazarte and Garcia‐Ruiz, 2011).

#### Gel made of sodium metasilicate

Commercial sodium metasilicate stock solution (Na_2_SiO_3_, ρ = 1.39 g ml^−1^, Cat. No. 33,844‐3, Aldrich) was diluted with water to prepare a working solution with a density of 1.06 g ml^−1^ using the relationship *V*_SS_ = (0.06 *V*_*a*_)/(1.39–*ρ* ^*T*^), where *V*_SS_ is the volume of stock solution required to prepare a final volume *V*_*a*_ and *ρ* ^*T*^ is the density of water at temperature *T* (Moreno *et al*., 1999). The sodium hydroxide contained in this sodium metasilicate solution (ρ = 1.06 g ml^−1^) was used to neutralize a 1 M acetic acid solution in order to obtain a monosilicic acid (H_4_SiO_4_) solution that polymerizes as a polysiloxane hydrogel. The same chemical gel can be prepared by the hydrolysis of either tetramethyl orthosilicate (TMOS) or tetraethyl orthosilicate (TEOS) as described elsewhere (Robert and Lefaucheux, 1988). Protection of skin and eyes is recommended during the handling of siloxanes as they are corrosive liquids. Glassware must be thoroughly rinsed with ethanol prior to cleaning them with water. As previously mentioned for agarose, the reactants can be incorporated to the silica gel by first diffusing them through the gel. The second layer, free of reactants, is then put in a sandwich configuration. Finally, the synthesis of these sulfides (located in the third gel layer of these heavy metals: Pb^2+^, Hg^2+^ and Cd^2+^) will occur by counter‐diffusion processes in the sandwich gel layer.

## Conflict of interest

The authors declare that they have no competing interest.

## Supporting information


**Table S1.** Concentration of Pb^2+^, Hg^2+^ or Cd^2+^ that resisted species *Candida*.
**Table S2.** Elements detected in sample fluorescence spectra using 16 KeV excitation energy.
