# Supplementary material for: Biosynthesis of micro‐ and nanocrystals of Pb (II), Hg (II) and Cd (II) sulfides in four Candida species: a comparative study of in vivo and in vitro approaches
Source: Microb Biotechnol. 2017 Jan 16;10(2):405–24. doi: 10.1111/1751-7915.12485 (PMC5328821; doi:10.1111/1751-7915.12485)
Supplement: Supplementary file 1 — Table S1. Concentration of Pb2+, Hg2+ or Cd2+ that resisted species Candida. Table S2. Elements detected in sample fluorescence spectra using 16 KeV excitation energy. [file MBT2-10-405-s001.docx]

**Table S1.** Concentration of Pb^2+^, Hg^2+^ or Cd^2+^ that resisted species *Candida.*

| **Specie** | **Concentration [mM]** | | |
| --- | --- | --- | --- |
|  | **Pb^2+^** | **Hg^2+^** | **Cd^2+^** |
| ***C. albicans*** | **2.0** | **2.0** | **2.0** |
| ***C. glabrata*** | **2.0** | **1.0** | **2.0** |
| ***C. krusei*** | **2.0** | **1.0** | **2.0** |
| ***C. parapsilosis*** | **2.0** | **1.0** | **2.0** |

**Table S2. Elements detected in sample fluorescence spectra using 16 KeV excitation energy.**

|  | *C. albicans* | *C. glabrata* | *C. krusei* | *C. parapsilosis* |
| --- | --- | --- | --- | --- |
| **Capillary Blank** | [Ar], K, Fe, [Cu], **Zn**, [Pb], [Br] | | | |
| **Pb Loaded** | [Ar], [K], [Fe], Zn, **Pb** | | | |
| **Hg Loaded** | [Ar], [K], [Fe], Zn, **Hg** | | | |
| **Loop Blank** | [Ar], Fe, [Cu], [Br] | | | |
| **Cd Loaded** | [Mn], S, [Ar], [K], Fe, [Br], Ca, **Zn**, **Cd** | | | |

Cd samples have been mounted on a Kapton loop with N-Paraton oil, while other samples were packed in borosilicate capillaries (elements in square parentheses were present as traces and could be due to environmental contamination – bolded symbols refer species estimated as more abundant from elemental analysis and, qualitatively, from fluorescence intensities).
